# Influence of different peritoneal dialysis fluids on the in vitro activity of fosfomycin against *Escherichia coli*, *Staphylococcus aureus*, *Staphylococcus epidermidis*, *and Pseudomonas aeruginosa*

**DOI:** 10.1007/s10096-018-3221-y

**Published:** 2018-03-15

**Authors:** Manuel Kussmann, Stefan Hauer, Petra Pichler, Gottfried Reznicek, Heinz Burgmann, Wolfgang Poeppl, Markus Zeitlinger, Martin Wiesholzer

**Affiliations:** 1 Department of Internal Medicine I, Division of Infectious Diseases and Tropical Medicine, Medical University Vienna, Vienna, Austria; 2 Department of Internal Medicine I, University hospital St. Poelten, Karl Landsteiner University of Health Sciences, St. Poelten, Austria; 3 Department of Pharmacognosy, University of Vienna, Vienna, Austria; 4 Military Medical Cluster East, Austrian Armed Forces, Vienna, Austria; 5 Department of Clinical Pharmacology, Medical University Vienna, Waehringerguertel 18-20, A-1090 Vienna, Austria

**Keywords:** Antibiotics, Infection, Peritoneal dialysis solutions, Peritonitis, Time-kill curves

## Abstract

Peritonitis is still the main infectious complication among patients on peritoneal dialysis. For treatment of peritoneal dialysis-related peritonitis, the intraperitoneal administration of antibiotics admixed to peritoneal dialysis fluids (PDFs) should be preferred. However, the influence of diverse PDFs on the activity of frequently used antibiotics has been investigated insufficiently. Thus, the present study set out to investigate the in vitro activity of fosfomycin against *Escherichia coli*, *Pseudomonas aeruginosa*, *Staphylococcus epidermidis*, and *Staphylococcus aureus* in commercially available PDFs. Time-kill curves in four different PDFs (Dianeal®, Extraneal®, Nutrineal®, and Physioneal®) were performed over 24 h with two different concentrations of fosfomycin (150 and 400 mg/L) and without antibiotics as control. Cation-adjusted Mueller Hinton broth (CA-MHB) was used as a comparator solution. In blank PDFs, bacterial growth of each organism evaluated was reduced when compared to CA-MHB. For *S. aureus* in blank Physioneal®, a reduction under the limit of detection was observed within 24 h. The activity of fosfomycin was reduced in all PDFs when compared to CA-MHB except for *P. aeruginosa* in Nutrineal® where the activity of fosfomycin was increased when investigated at 400 mg/L. Against *E.coli*, bactericidal activity was demonstrated in Extraneal®, Nutrineal®, and Physioneal®. Fosfomycin resistance (MIC > 1024 mg/L) was observed for *P. aeruginosa* in CA-MHB at both concentrations and in Nutrineal® at 150 mg/L. Fosfomycin is active in PDFs particularly against the frequently isolated enterobacterium *E. coli*. The choice of the respective PDF considerably influences the microbiological outcome in vitro. Further studies are warranted to investigate the clinical relevance of these findings.

## Introduction

Peritoneal dialysis-related peritonitis (PDRP) remains a serious infectious complication among patients undergoing peritoneal dialysis (PD) and is the most common isolated cause of a modality switch to hemodialysis [1, 2]. The pathogens most frequently isolated from patients with PDRP are Staphylococci among Gram-positive and *Escherichia coli* as well as *Pseudomonas aeruginosa* among Gram-negative organisms [3–5]. Therefore, according to the most recent guidelines of the International Society for Peritoneal Dialysis (ISPD), empiric antimicrobial regimens should cover Gram-positive and Gram-negative pathogens [1]. Due to higher drug concentrations at the target site, improved feasibility and compliance, the recommended route for antibiotic administration in patients with PDRP is the intraperitoneal (IP) application with the drug admixed to peritoneal dialysis fluids (PDFs) [1, 6].

The emergence of drug-resistant bacteria, particularly multi-resistant Gram-negative organisms and Staphylococci, complicates the treatment of PDRP and highlights the need for new therapeutic regimens [7]. Fosfomycin demonstrates good in vitro and in vivo activity against several Gram-positive and Gram-negative organisms, excellent tissue distribution and tolerability [8–10]. A recent pharmacokinetic study in patients on automated PD (APD) demonstrated sufficiently high dialysate and serum concentrations adequate for treatment of PDRP as well as systemic infections after IP administration of a single dose of 4 g fosfomycin [11]. However, the influence of PDFs on the antimicrobial activity of fosfomycin has not been evaluated. Thus, the present study set out to investigate the in vitro activity of fosfomycin in different commercially available PDFs against four of the most common pathogens of PDRP.

## Materials and methods

### Bacterial strains and susceptibility tests

*E. coli* (ATCC 25922), methicillin-resistant *S. aureus* (MRSA) (ATCC 33592), *S. epidermidis* (DSM 20044), and *P. aeruginosa* (ATCC 27853) were used in this study. Minimal inhibitory concentrations (MICs) of fosfomycin were determined in triplicates by Etests (Biomerieux Deutschland GmbH, Nurtingen, Germany) and broth microdilution in cation-adjusted Mueller Hinton broth (CA-MHB) supplemented with glucose-6-phosphate (Sigma-Aldrich, Vienna, Austria): 1 μg/mL for *E. coli*, 6 μg/mL for MRSA, 0.38 μg/mL for *S. epidermidis*, and 6 μg/mL for *P. aeruginosa*. After each experiment, MICs of the obtained colonies were determined in duplicates by Etests to detect the potential emergence of antimicrobial resistance.

### Antibiotics and growth media

For this study, four different commercially available PDFs, namely Dianeal® PDG4 (1.36% glucose), Extraneal® (75 g/2000 ml icodextrin), Nutrineal® PD4 (1.1% amino acids), and Physioneal® 40 (2.27% glucose) were used. To simulate the physiological pH after a 4–6 h intraperitoneal dwell, the pH of all PDFs was adjusted to 7.30–7.50 with NaOH [12–14]. CA-MHB (pH 7.4) was used as comparator solution. Fosfomycin was obtained in form of dry powder (Fosmicin®, Meiji Seika Pharma Co., Ltd., Tokyo, Japan), diluted in sterile distilled water, and stored at − 80 °C until use.

### Time-kill assays

Time-kill curves with four different bacteria were performed in the diverse PDFs and in CA-MHB comparator solution. Bacteria were grown overnight on 5% sheep blood agar plates at 37 °C, suspended in 0.9% sterile saline to a concentration equivalent to a 0.5 McFarland standard, and diluted 1:100 in PDFs or CA-MHB to obtain final inoculums. Ten milliliter tubes were incubated on an orbital shaker for 24 h at 37 °C. Two hours after start, fosfomycin was added to achieve final concentrations of 150 or 400 mg/L representing intraperitoneal concentrations obtained in patients on APD at 6 and 12–14 h after a single dose of 4 g fosfomycin IP [11].

Samples were taken at − 2, 0 (immediately before antibiotic addition), 2, 6, 10, and 24 h.

Control assays without fosfomycin were run in all PDFs and in CA-MHB comparator solution. Bacterial counts were determined by using tenfold dilutions plated on 5% sheep blood agar plates which were incubated for 24 h at 37 °C. Time-kill curves for all bacteria at both concentrations of fosfomycin and for controls without fosfomycin were obtained by plotting log_10_ colony-forming units per milliliter versus time. Bactericidal activity was defined as a reduction of ≥ 3 log_10_ CFU/mL compared to initial inoculum (directly before antibiotic addition).

## Results

**Fig. 1:**
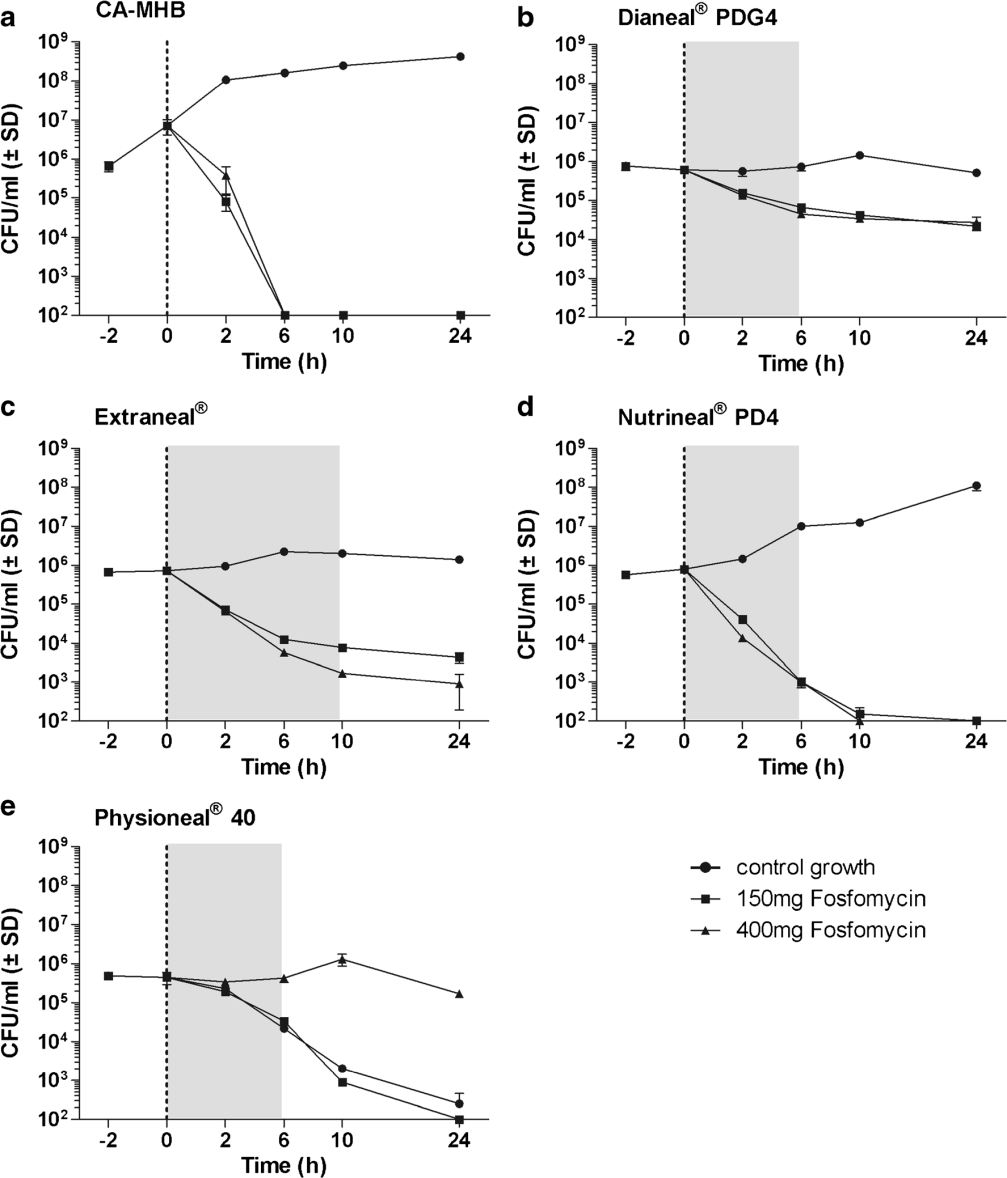
Data are presented as mean ± SD, *n* = 2. Time-kill curves with *E. coli* in CA-MHB comparator solution and in four different peritoneal dialysis fluids at concentrations of 150 and 400 mg/L fosfomycin. The dotted line shows the time of antibiotic addition which was done directly after bacterial counts. The area in gray represents the time of dwell periods frequently used in clinical practice. **a** CA-MHB, **b** Dianeal® PDG4, **c** Extraneal®, **d** Nutrineal® PD4, **e** Physioneal® 40

**Fig. 2:**
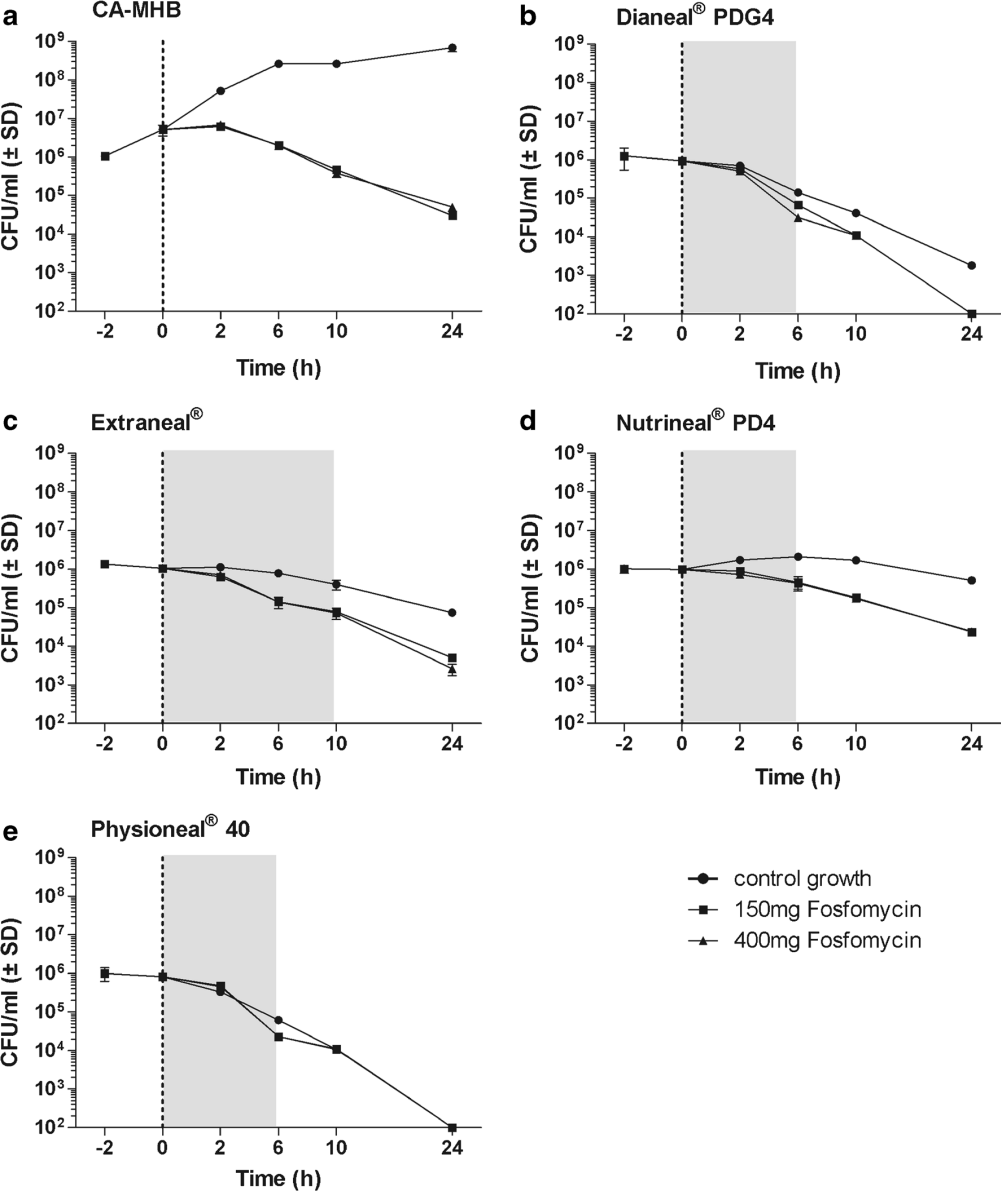
Data are presented as mean ± SD, *n* = 2. Time-kill curves with MRSA in CA-MHB comparator solution and in four different peritoneal dialysis fluids at concentrations of 150 and 400 mg/L fosfomycin. The dotted line shows the time of antibiotic addition which was done directly after bacterial counts. The area in gray represents the time of dwell periods frequently used in clinical practice. **a** CA-MHB, **b** Dianeal® PDG4, **c** Extraneal®, **d** Nutrineal® PD4, **e** Physioneal® 40

**Fig. 3:**
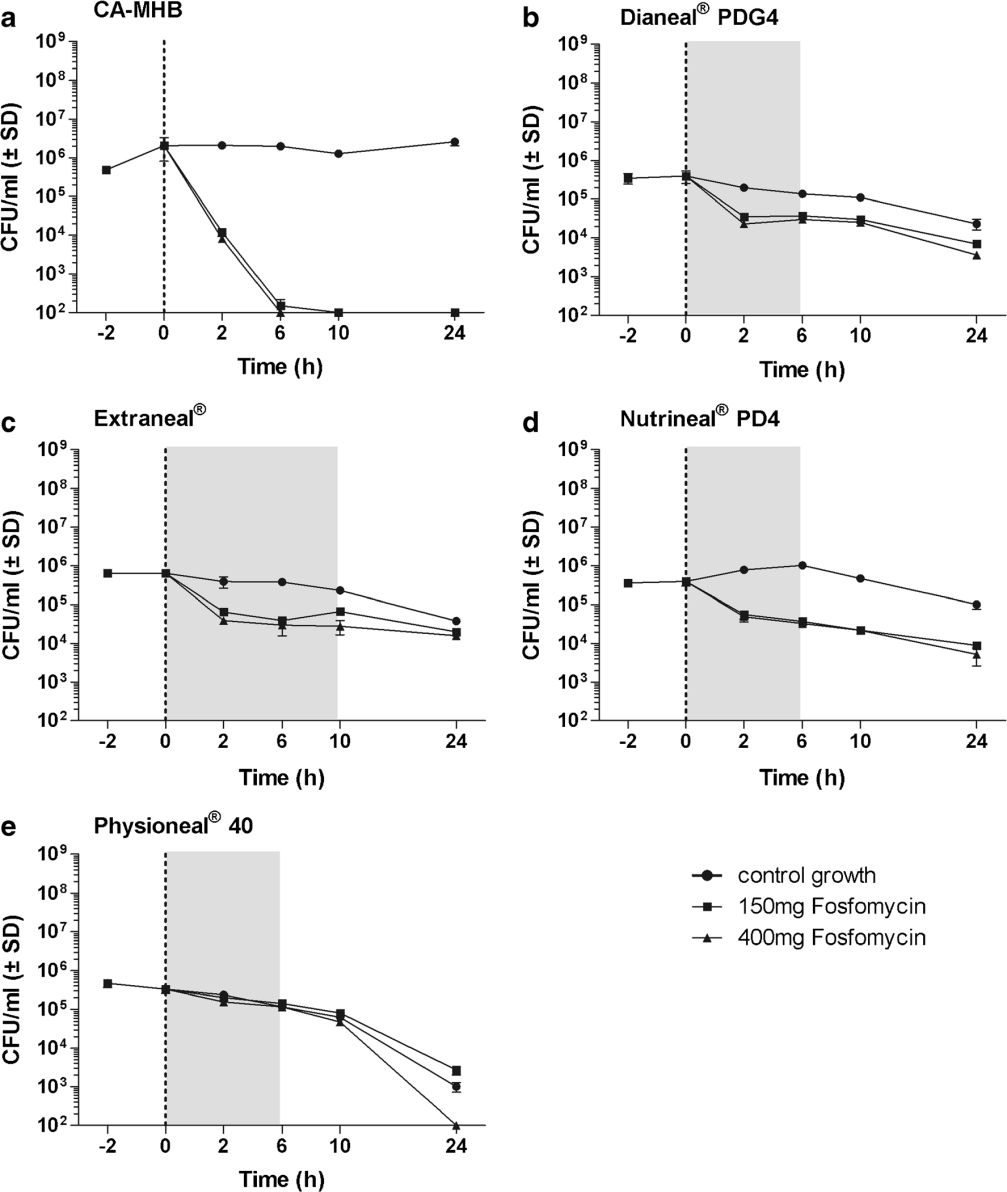
Data are presented as mean ± SD, *n* = 2. Time-kill curves with *S. epidermidis* in CA-MHB comparator solution and in four different peritoneal dialysis fluids at concentrations of 150 and 400 mg/L fosfomycin. The dotted line shows the time of antibiotic addition which was done directly after bacterial counts. The area in gray represents the time of dwell periods frequently used in clinical practice. **a** CA-MHB, **b** Dianeal® PDG4, **c** Extraneal®, **d** Nutrineal® PD4, **e** Physioneal® 40

**Fig. 4:**
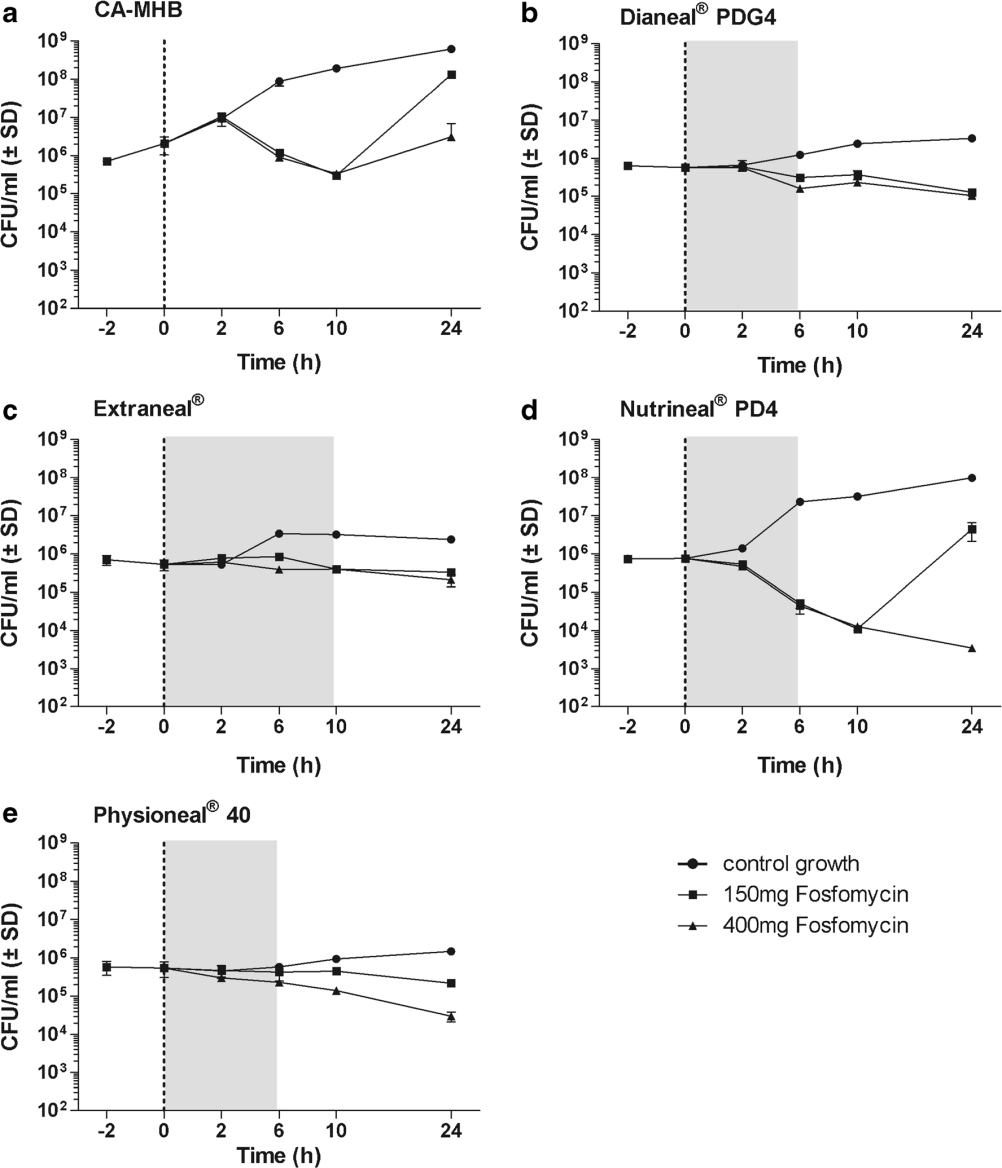
Data are presented as mean ± SD, *n* = 2. Time-kill curves with *P. aeruginosa* in CA-MHB comparator solution and in four different peritoneal dialysis fluids at concentrations of 150 and 400 mg/L fosfomycin. The dotted line shows the time of antibiotic addition which was done directly after bacterial counts. The area in gray represents the time of dwell periods frequently used in clinical practice. **a** CA-MHB, **b** Dianeal® PDG4, **c** Extraneal®, **d** Nutrineal® PD4, **e** Physioneal® 40

Time-kill curves are outlined in Figs. 1, 2, 3, and 4. Compared to CA-MHB comparator solution, a reduction of bacterial growth or even bacterial killing could be demonstrated in blank PDFs for all organisms evaluated. In particular for *S. aureus*, a reduction of bacterial counts under the limit of detection was demonstrated in blank Physioneal® (Fig. 2).

The mean reduction in log_10_ colony-forming units per milliliter of *E. coli*, *S. aureus*, *S. epidermidis*, and *P. aeruginosa* after an incubation of 24 h with 400 mg/L fosfomycin were as follows: 4.86, 2.01, 4.31, and − 0.18 in CA-MHB; 1.35, 3.97, 2.04, and 0.74 in Dianeal®; 3.04, 2.60, 1.60, and 0.40 in Extraneal®; 3.90, 1.61, 1.88, and 2.34 in Nutrineal®; and 3.65, 3.92, 3.52, and 1.26 in Physioneal®, respectively.

When compared to CA-MHB, fosfomycin showed a reduced and/or delayed antimicrobial activity against all organisms, in all PDFs investigated except for *P. aeruginosa* in Nutrineal®. For *P. aeruginosa* in Nutrineal®, fosfomycin at 400 mg/L demonstrated increased bacterial killing, but at low concentration (150 mg/L), a rebound of bacterial counts was observed between 12 and 24 h (Fig. 4). The MICs obtained before and after each time-kill assay revealed an induced antimicrobial resistance against fosfomycin for *P. aeruginosa* in CA-MHB (> 1024 mg/L) at both fosfomycin concentrations investigated (150 and 400 mg/L) and in Nutrineal® (> 1024 mg/L) at low concentration (150 mg/L), but not in any other PDF or for any other organism. Bactericidal activity of fosfomycin, defined as a reduction of ≥ 3 log_10_ CFU/mL, was demonstrated against *E. coli* in Extraneal®, Nutrineal®, and Physioneal® at a concentration of 400 mg/L and in Nutrineal® and Physioneal® at both concentrations evaluated (Fig. 1) but not against any other organism in PDFs (Figs. 2, 3, and 4). In clinical routine Dianeal®, Nutrineal® and Physioneal® are commonly administered IP for short dwells lasting 4–6 h, whereas Extraneal® is applied for longer dwells. Therefore, the mean differences in colony-forming units per milliliter between PDFs with fosfomycin and PDF controls of all bacteria tested, 6 h (for Dianeal®, Nutrineal®, and Physioneal®) and 10 h (for Extraneal®) after addition of fosfomycin, are presented for each PDF in Table 1.

**Table 1:** Mean differences in log_10_ colony-forming units per milliliter between specific peritoneal dialysis fluids (PDFs) with fosfomycin at two different concentrations and blank PDFs at time points representing duration of dwells (6 h for Dianeal® PDG4, Extraneal®, Nutrineal® PD4, and Physioneal® 40; 10 h for icodextrin-based Extraneal®)

	Antibiotics	Mean difference of viable microorganisms compared to PDF control without antibiotics in log_10_ colony-forming units per milliliter (*p* value)				
		Dianeal® PDG4 (6 h)	Extraneal® (6 h)	Extraneal® (10 h)	Nutrineal® PD4 (6 h)	Physioneal® 40 (6 h)
*Escherichia coli*	Fosfomycin 150 mg/L	1.04	2.25	2.41	4.00	1.30
	Fosfomycin 400 mg/L	1.22	2.59	3.07	4.00	1.10
*Staphylococcus aureus*	Fosfomycin 150 mg/L	0.33	0.74	0.70	0.67	0.42
	Fosfomycin 400 mg/L	0.66	0.75	0.74	0.69	0.42
*Staphylococcus epidermidis*	Fosfomycin 150 mg/L	0.57	0.99	0.54	1.44	−0.09
	Fosfomycin 400 mg/L	0.66	1.10	0.91	1.49	0.00
*Pseudomonas aeruginosa*	Fosfomycin 150 mg/L	0.59	0.60	0.90	2.65	0.13
	Fosfomycin 400 mg/L	0.88	0.94	0.90	2.72	0.40

## Discussion

In the present study, the growth of *E. coli*, *S. aureus*, *S. epidermidis*, and *P. aeruginosa* was reduced in PDFs compared to conventional growth medium. For Staphylococci, even bactericidal killing could be observed in glucose-containing solutions but not in any other PDF or for any other bacteria. When compared to CA-MHB, fosfomycin demonstrated a delayed and/or reduced antimicrobial activity in PDFs, except for *P. aeruginosa* in Nutrineal® where fosfomycin showed an increase of bacterial killing when applied at high concentration of 400 mg/L. At lower concentration of 150 mg/L as well as in CA-MHB, *P. aeruginosa* developed a fosfomycin resistance with a MIC of > 1024 mg/L.

No or only little activity of fosfomycin was demonstrated against *S. aureus* and *S. epidermidis* when compared to PDF controls which might be mainly attributed to the high antibacterial activity of the glucose-containing PDFs Physioneal® and Dianeal® on the one hand and to the reduced activity of fosfomycin in Extraneal® and Nutrineal® on the other hand. In contrast, fosfomycin demonstrated excellent activity against *E. coli* when tested in Nutrineal®, Physioneal®, and Extraneal® and against *P. aeruginosa* in Nutrineal®. Altogether, these findings highlight the impact of differently composited PDFs on bacterial growth and on the activity of antimicrobial drugs.

In addition, the increased activity of high dose fosfomycin (400 mg/L) in Nutrineal®, the PDF, which demonstrated the highest proliferation rate of *P. aeruginosa* when investigated as blank solution, supports the assumption that the bacteriostatic effect of PDFs might be the cause of reduced antimicrobial activity in PDFs in vitro [13–16].

In contrast to in vitro studies highlighting the relevance of a specific PDF on the microbiological outcome of several antimicrobial agents, clinical studies on the management of PDRP so far neglected this potential parameter. According to the data of several clinical trials and a recently published meta-analysis, most of the studies do not report the respective PDFs used [17]. Moreover, as the majority of these studies included only a small study population or are of poor quality with incongruent dosing regimens and definitions, it is currently not possible to determine a superior antimicrobial agent or antibiotic class for treatment of PDRP [17].

One possible factor contributing to the reduced activity of fosfomycin observed in vitro could be insufficient stability in certain PDFs. However, a recent study investigated the compatibility of fosfomycin with Extraneal®, Nutrineal®, Physioneal® 1.36%, and Physioneal® 2.27% over 14 days at 6 and 25 °C as well as over 24 h at 37 °C. The relative drug concentrations at the end of each storage period (14 days at 6 and 25 °C; 24 h at 37 °C) ranged between 94 and 104% of the initial fosfomycin concentration. Thus, defined as a drug decomposition of ≤ 10% of the initial concentration, fosfomycin was shown to be stable in all PDFs and at each storage condition investigated over the whole study period [18].

For many antimicrobials including aminoglycosides, fluoroquinolones, and some beta-lactams, a pH dependence with a reduced activity in a low pH milieu is known [19–22]. Whereas, Physioneal® displays a physiological pH after mixing of the two chambers, the pH values of Dianeal®, Extraneal®, and Nutrineal® range between 4 to 6 and thus, could reduce the activity of certain antimicrobials in vivo. Some data obtained from PDFs retained from patients indicate that after an IP dwell of 4 to 6 h, a physiological pH is reached. However, no information exists about the velocity of this process [12, 13]. Thus, in accordance to previous studies, in the present study, low-pH PDFs were adjusted to a pH of 7.30–7.50 at the beginning of each experiment [13, 14]. For fosfomycin, however, a recent study by Fedrigo et al. demonstrated that its activity against two Enterobacteriaceae was even improved by acidification of the growth medium [23].

The concentrations of fosfomycin used in the present study were chosen to mimic the clinical situation as closely as possible. In a pharmacokinetic study, including eight non-infected patients undergoing APD, Tobudic et al. demonstrated high dialysate and serum levels after IP administration of 4 g fosfomycin, sufficient for treatment of PDRP and systemic infections. After a single dose of fosfomycin IP, dialysate drug concentrations were shown to remain above 400 and 150 mg/L over 6 h and 12–14 h, respectively. In contrast, after IV administration of 4 g fosfomycin, insufficient concentrations were observed in the peritoneal dialysis solution [11].

Thus, based on the pharmacokinetic data published by Tobudic et al. and on the in vitro activity observed in the present study, intraperitoneal fosfomycin might be an effective treatment option for patients with PDRP.

In conclusion, fosfomycin was shown to be highly active against the frequently isolated enterobacterium *E. coli* in commercially available PDFs and, thus, may serve as an effective and well tolerated alternative for empiric and/or targeted treatment of PDRP. The choice of differently composited PDFs might considerably influence bacterial growth as well as the antimicrobial activity of fosfomycin and might thus have a relevant impact on the clinical outcome. Further studies are warranted to investigate the clinical relevance of these findings.
